# Treatable Traits in Pediatric Interstitial Lung Diseases: Bridging the Gap to Tailored Therapeutics

**DOI:** 10.3390/jcm14228190

**Published:** 2025-11-19

**Authors:** Giuseppe Fabio Parisi, Maria Papale, Giulia Pecora, Santiago Presti, Monica Tosto, Salvatore Leonardi

**Affiliations:** Pediatric Respiratory Unit, Department of Clinical and Experimental Medicine, San Marco Hospital, University of Catania, 95121 Catania, Italy; m.papale@policlinico.unict.it (M.P.); giupec87@hotmail.it (G.P.); santiago.presti@policlinico.unict.it (S.P.); monitosto@gmail.com (M.T.); leonardi@unict.it (S.L.)

**Keywords:** pediatric interstitial lung disease, treatable traits, personalized medicine, genetic disorders, pulmonary hypertension, immune dysregulation, multidisciplinary care

## Abstract

Pediatric interstitial lung diseases (chILD) are a diverse and complex group of rare but impactful disorders characterized by heterogeneous etiologies and variable clinical courses. Traditional diagnosis-based management often delays targeted treatment, underscoring the need for a more precise therapeutic approach. The “treatable traits” framework, originally developed in adult respiratory medicine, offers a novel paradigm for personalized care by focusing on identifying and modifying discrete, clinically relevant features in each child. This narrative review synthesizes existing evidence and expert consensus to define key treatable traits in pediatric ILD, encompassing genetic and surfactant dysfunction, immune dysregulation, pulmonary hypertension, hypoxemia, aspiration, growth deficits, and environmental exposures. For each trait, we describe diagnostic pathways—including genetic testing, bronchoalveolar lavage, imaging, and functional assessments—and outline targeted management strategies. The implementation of a trait-based approach necessitates multidisciplinary collaboration, standardized protocols, and ongoing research to validate biomarkers and optimize therapies. By adopting this personalized strategy, clinicians can improve early diagnosis, tailor interventions, and potentially alter disease trajectories. Our discussion highlights the current limitations and future priorities, emphasizing the importance of pediatric-specific studies and international networks to fully realize the promise of precision medicine in pediatric ILD.

## 1. Introduction

Pediatric interstitial lung diseases (chILD) encompass a broad and diverse group of diffuse parenchymal lung disorders affecting infants, children, and adolescents. Although individually rare, collectively they represent a significant clinical challenge due to their heterogeneity, potential for morbidity, and impact on growth, neurodevelopment, and quality of life [1]. These conditions can present in infancy with respiratory failure, persistent cough, hypoxemia, or restriction, and in older children with progressive, insidious pulmonary decline. The complexity of chILD is heightened by overlapping clinical, radiological, and histopathological features that often blur the distinction between different etiologies, including developmental anomalies, genetic surfactant dysfunction, inflammation, infections, aspiration, and systemic diseases [2,3].

One of the main hurdles in managing pediatric interstitial lung diseases is their diagnostic and therapeutic diversity. Limited pediatric-specific evidence, rarity of individual entities, and overlapping phenotypes make diagnosis challenging and delay specific treatment. Traditional approaches based on syndrome classification or broad diagnostic categories sometimes fall short in guiding precise, effective interventions, leading to prolonged empirical therapies with potential adverse effects [4].

The “treatable traits” paradigm, initially developed in adult respiratory medicine, offers an innovative approach to overcome these challenges. It emphasizes identifying discrete, clinically meaningful, and modifiable features within each patient—traits that can be biologically, physiologically, or clinically defined—and directing targeted treatments towards them. This approach shifts focus from static diagnostic labels to dynamic, personalized management strategies that can be tailored to the evolving clinical picture [5].

Applying the treatable traits framework to pediatric ILD holds great promise. Many traits—such as pulmonary hypertension, surfactant failure, immune dysregulation, or aspiration—are modifiable and amenable to specific therapeutic interventions. Early recognition through advanced diagnostics (genetic testing, bronchoalveolar lavage, imaging, biomarkers) can facilitate targeted therapy, potentially improving outcomes and quality of life. Moreover, structuring care around traits could foster more efficient research, data sharing, and registry development by grouping patients based on actionable features rather than rare diagnoses alone [6].

This review aims to present a conceptual framework for identifying and managing treatable traits in pediatric ILD. Due to the wide array of potential factors, traits are grouped based on their primary nature: biologic (e.g., genetic, surfactant dysfunction), physiologic (e.g., pulmonary hypertension, hypoxemia), and comorbid (e.g., aspiration, nutritional deficits). This structure is intended to facilitate a practical approach to identifying and addressing key modifiable features in children with ILD.

## 2. Materials and Methods

This narrative review was conducted through a comprehensive literature search to identify and synthesize current evidence regarding treatable traits in chILD. To ensure the review reflects the latest available data, the literature search was performed up to August 2025. Given the broad and heterogeneous nature of the topic, a scoping review framework was employed, facilitating an inclusive mapping of diagnostic and therapeutic strategies relevant to trait-based management in chILD. A thorough search of multiple electronic databases, including MEDLINE via PubMed, Embase, the Cochrane Library, and Web of Science, was performed. The search strategy was developed in collaboration with information specialists and combined controlled vocabulary (such as MeSH and Emtree terms) with free-text keywords. Keywords and search strings included terms like “pediatric interstitial lung disease,” “chILD,” “diffuse lung disease,” “surfactant dysfunction,” “pulmonary hypertension,” “immune dysregulation,” “biomarkers,” “bronchoalveolar lavage,” “targeted therapy,” “treatable traits,” among others. To capture the most recent developments, no date restrictions were applied initially; the search was limited to articles published up to August 2025.

Inclusion criteria focused on articles that provided insights into diagnostic approaches, biomarkers, or therapeutic interventions targeting specific, modifiable traits in children with confirmed or suspected interstitial lung disease. This encompassed original research, case series, case reports, clinical guidelines, and expert consensus publications that contributed to understanding trait identification or management strategies. Particular emphasis was placed on literature offering practical diagnostic pathways and evidence-based or promising targeted therapies.

Studies limited strictly to adult populations were excluded unless their findings were directly applicable or transferable to the pediatric context. Articles lacking specific information on diagnostic procedures or therapeutic interventions aimed at modifiable traits were omitted. To ensure rigor and reduce bias, article selection was performed independently by two reviewers, with disagreements resolved through discussion or third-party adjudication.

Data extraction was performed using a structured form capturing details about each trait, including definitions and prevalence, diagnostic modalities, biomarkers, therapeutic options, evidence levels, and clinical outcomes. The evidence synthesis was predominantly narrative, focusing on practical, clinically relevant insights that could inform a trait-guided management framework. The review also acknowledges existing gaps in pediatric-specific evidence and emphasizes the importance of ongoing research to refine the application of treatable traits in pediatric ILD.

In the preparation of this manuscript, generative artificial intelligence (GenAI) tools were not used for generating the core content, study design, data collection, analysis, or interpretation. Superficial editing tasks such as grammar, spelling, punctuation, and formatting may have been assisted by AI-based editing tools (Chat GPT v. 5.1), but these do not require disclosure. All substantive content, including the framing of the research methodology and synthesis of evidence, was developed independently by the authors.

## 3. Results

This review identified a wide array of potential treatable traits in pediatric interstitial lung diseases, encompassing genetic, immunologic, vascular, infectious, and structural components. The evidence drawn from pediatric case series, expert guidelines, and translational studies highlights the diverse and interconnected nature of these features. Below, we categorize and describe the main traits, their diagnostic pathways, and the current therapeutic options supported by available evidence.

### 3.1. Key Treatable Traits in Pediatric ILD

#### 3.1.1. Genetic and Surfactant Dysfunction Traits

Genetic mutations impacting surfactant protein genes constitute a critical and increasingly recognized treatable trait in pediatric interstitial lung disease. These disorders often present very early in life with severe respiratory compromise, such as neonatal respiratory distress syndrome that persists beyond the neonatal period, or with overt interstitial abnormalities observable on high-resolution imaging during infancy or early childhood. The severity and course of disease are highly variable and are largely dictated by the specific genetic defect and its impact on surfactant production, processing, or transport [7,8,9].

The most frequently implicated genes include surfactant protein B (SFTPB), surfactant protein C (SFTPC), and ATP-binding cassette transporter A3 (ABCA3). Mutations in these genes disrupt the synthesis, assembly, or secretion of surfactant components, leading to impaired alveolar stability, recurrent atelectasis, and progressive fibrosis. Certain mutations, particularly in SFTPB, are associated with lethal neonatal respiratory distress, whereas others, such as SFTPC mutations, may present later in childhood with a broader range of phenotypes, including interstitial pneumonitis, fibrosis, or restrictive lung disease [10].

The diagnosis relies on a combination of genetic testing and functional analysis. Important genes to consider for surfactant polymorphism testing, which impact surfactant production, structure, or function, include SFTPA1, SFTPA2, SFTPB, SFTPC, SFTPD, and ABCA3. Since these disorders can have overlapping clinical presentations, a high index of suspicion is necessary, especially in children with persistent interstitial infiltrates, early respiratory failure, or family history suggestive of inherited lung disease. Next-generation sequencing panels, targeting known surfactant pathway genes, are the primary diagnostic modality. When a causative mutation is identified, additional testing, such as surfactant protein immunohistochemical staining or electron microscopy of lung biopsy specimens, can provide further information regarding the structural abnormalities associated with specific mutations [11,12].

The management of surfactant-related ILD remains primarily supportive. Lung-protective oxygen therapy, nutritional support, and meticulous respiratory care are essential to optimize quality of life. Mechanical ventilation may be necessary during acute exacerbations or in severe cases, and lung transplantation remains the definitive option for irreversible end-stage disease [2,3].

Currently, specific pharmacological treatments targeting the underlying genetic defects are limited. Nonetheless, several promising avenues are under active investigation. Recombinant surfactant therapy has shown benefit in some cases, particularly of SFTPB deficiency, although its efficacy is variable [12]. Newer approaches include small molecules designed to enhance surfactant processing, molecular chaperones to assist correct folding of mutant proteins, and gene therapy techniques aiming to replace or repair defective alleles. While these targeted therapies are not yet part of routine clinical practice, ongoing research and clinical trials hold the potential to transform the management landscape [12,13].

Early diagnosis of surfactant gene mutations not only helps guide clinical management but also provides critical information for family counseling, including recurrence risks and options for prenatal diagnosis. Recognizing this trait promptly can facilitate timely intervention, better prognostication, and appropriate consideration for advanced therapies such as lung transplantation, ultimately improving survival and quality of life for affected children.

#### 3.1.2. Active Airway Inflammation and Immune-Mediated Traits

In chILD, immune-mediated and inflammatory traits are central components that often drive disease activity and progression. Children presenting with subacute or chronic pneumonitis, particularly those with persistent symptoms despite supportive care, warrant evaluation for immune dysregulation as a treatable trait. These presentations may include recurrent or refractory infiltrates, ground-glass opacities, nodular patterns on imaging, and histopathologic features of cellular infiltrates, granulomas, or organizing pneumonia [14].

The identification of immune-mediated traits heavily relies on bronchoalveolar lavage (BAL), which often reveals a lymphocytic predominance—typically increased CD4+ and CD8+ T-cell populations—suggesting ongoing alveolar inflammation. Elevated levels of inflammatory biomarkers, such as interleukin-6 (IL-6), tumor necrosis factor-alpha (TNF-α), and other cytokines, may further support this diagnosis. In certain cases, a lung biopsy is performed, especially when the clinical picture is complex or when non-invasive studies are inconclusive. Histopathological examination may demonstrate lymphoid aggregates, granulomas, or organizing pneumonia, which reinforce the diagnosis of immune-driven pathology [15,16]. In cases of chILD associated with systemic inflammatory or autoimmune conditions, biological therapies targeting specific cytokines, such as anti-TNF alpha or anti-IL-6 agents, may be considered. Biologic therapies hold promise for select cases of pediatric ILD, particularly those associated with systemic inflammatory conditions or specific monogenic immune disorders. Agents such as anti-TNFα (e.g., infliximab, etanercept), anti-IL-6 (e.g., tocilizumab), and other cytokine inhibitors are being explored. However, their use in chILD remains largely off-label and requires careful consideration of potential risks, including increased susceptibility to infection and paradoxical inflammatory responses. Further pediatric-specific studies are essential to define the efficacy and safety of these agents and to identify biomarkers that can predict treatment response [17,18].

Diagnosis of immune-mediated traits involves a comprehensive assessment that combines cellular analysis of BAL fluid, immunoserology (including autoimmune panels, immunoglobulin levels, and specific autoantibodies), and, in selected cases, genetic testing for known immune dysregulation syndromes. Differential diagnoses such as autoimmune connective tissue diseases, hypersensitivity pneumonitis, and sarcoidosis should be systematically evaluated [2,3,17].

Management strategies focus on suppressing pathological immune activation while preserving the child’s overall immune competence. Corticosteroids are frequently used as first-line therapy, aiming to reduce alveolar inflammation rapidly. Depending on disease severity and response, additional immunomodulators, such as mycophenolate mofetil, azathioprine, or calcineurin inhibitors, may be employed to facilitate steroid-sparing effects or to target specific pathways involved in immune dysregulation [4].

Early recognition and timely initiation of targeted immunotherapy are associated with favorable outcomes, including stabilization or improvement of lung function, resolution of inflammation, and prevention of fibrosis. The trajectory of immune-mediated traits emphasizes the importance of serial clinical assessments, repeated BAL, and close monitoring of inflammatory markers to guide therapy adjustments and evaluate response [19].

Furthermore, the impact of antibiotic exposure on the respiratory microbiome also warrants consideration. Excessive or repeated antibiotic use, commonly encountered in children with chronic respiratory conditions, can disturb the delicate balance of the gut and lung microbiota. Disruption of the microbial ecosystem can lead to reduced microbial diversity, an increased risk of opportunistic infections, and potentially altered immune responses within the lung. Emerging evidence suggests that the gut-lung axis plays a significant role in modulating pulmonary inflammation and immunity, and disruptions in either microbial community may influence the pathogenesis and progression of chILD. Therefore, antibiotic stewardship and strategies aimed at restoring a healthy microbiota (e.g., probiotics, prebiotics) may be important considerations in the management of children with child [20,21].

The potential role of symbiotic supplementation (combining probiotics and prebiotics) in modulating lung health and reducing the risk of relapses and infections in children with chILD is an area of increasing interest. While direct evidence in the chILD population is currently limited, studies in other pediatric respiratory conditions, such as recurrent wheezing, asthma, and cystic fibrosis, have suggested that symbiotic supplementation can positively influence the gut-lung axis, potentially leading to improved immune function and reduced airway inflammation. Some studies have shown a trend towards decreased respiratory infections and a reduction in the frequency or severity of exacerbations with symbiotic use. The proposed mechanisms include modulation of the gut microbiota composition, enhancement of intestinal barrier function, and improved systemic immune responses. Given the potential for immune dysregulation to contribute to the pathogenesis of chILD, further research is needed to evaluate the efficacy and safety of symbiotic interventions in this population and to identify specific microbial profiles that may predict a favorable response [22,23,24].

Importantly, understanding the underlying immune process can sometimes reveal underlying systemic autoimmune or autoinflammatory syndromes, facilitating multidisciplinary management involving rheumatology, immunology, and pulmonology specialists. As knowledge evolves, the identification of specific immune biomarkers and molecular signatures promises to refine diagnostic accuracy and enable personalized immunomodulatory therapy, ultimately improving prognoses for affected children.

#### 3.1.3. Pulmonary Hypertension and Vascular Traits

Pulmonary hypertension (PH) is a significant and life-threatening vascular trait frequently observed in children with interstitial lung disease. It results from increased resistance within the pulmonary vasculature, secondary to vascular remodeling, hypoxic vasoconstriction, and inflammation, all of which may be driven or exacerbated by underlying lung pathology. The presence of PH complicates disease management, exacerbates right ventricular strain, and is associated with a poorer prognosis in pediatric ILD [25].

The early detection of PH involves a high index of suspicion in children with worsening respiratory symptoms, unexplained exercise intolerance, or echocardiographic signs suggesting elevated pulmonary pressures. Transthoracic echocardiography serves as the initial screening modality due to its non-invasive nature, allowing assessment of right ventricular size and function, pulmonary artery pressures, and indirect signs of elevated pulmonary vascular resistance. When echocardiographic findings are suggestive of PH or if clinical suspicion remains high despite non-diagnostic echocardiograms, right heart catheterization—considered the gold standard—is performed to confirm the diagnosis, accurately measure pulmonary artery pressures, and evaluate vascular resistance and cardiac output [26].

Once diagnosed, the management of pulmonary vascular traits involves a multidisciplinary approach integrating optimized pulmonary care with pulmonary vasodilator therapy. Pharmacologic agents such as phosphodiesterase type 5 (PDE5) inhibitors (e.g., sildenafil, tadalafil) and endothelin receptor antagonists (e.g., bosentan, ambrisentan) are the cornerstone of targeted therapy. These agents work by promoting vasodilation and reversing vasoconstriction, thereby reducing pulmonary artery pressures and improving right ventricular function [26,27].

While the evidence supporting the use of pulmonary vasodilators in pediatric ILD is limited and largely extrapolated from adult studies, clinical experience suggests early initiation of therapy can improve symptoms, exercise capacity, and hemodynamic parameters. However, caution is necessary, as vasodilation in the setting of active parenchymal inflammation or ventilation-perfusion mismatch may precipitate hypoxemia or exacerbate ventilation issues in some cases. Close monitoring with serial echocardiography and clinical assessments is essential to guide therapy modifications and assess response [28].

Ongoing research aims to better define the pediatric-specific benefits and risks of pulmonary vasodilators in children with ILD-associated PH. Future studies are needed to establish optimal timing, duration, and combinations of therapies, as well as to develop biomarkers for early detection and response assessment. Recognizing and treating pulmonary vascular traits promptly can significantly improve quality of life and survival, highlighting the importance of routine screening for PH in children with ILD, particularly those with progressive or refractory disease [29].

#### 3.1.4. Hypoxemia and Ventilatory Insufficiency Traits

Chronic hypoxemia and ventilatory inadequacy are prevalent traits in pediatric ILD, reflecting impaired alveolar gas exchange due to widespread parenchymal involvement, airway obstruction, or reduced ventilatory capacity. These traits critically influence disease severity, quality of life, and prognosis, necessitating systematic assessment and targeted intervention [30].

Identification and assessment of hypoxemia begins with pulse oximetry—both at rest and during activity—to detect resting or exertional oxygen desaturation. For more precise quantification, arterial blood gas (ABG) analysis provides O_2_ and CO_2_ partial pressures, confirming hypoxemia (commonly defined as PaO_2_ < 60 mm Hg) and assessing ventilatory efficiency via carbon dioxide levels. Pulmonary function tests (PFTs), including measurement of forced vital capacity (FVC), functional residual capacity (FRC), and diffusing capacity for carbon monoxide (DLCO), help delineate the extent of ventilatory compromise and gas exchange impairment [31].

Management strategies are tailored to the severity of hypoxemia and ventilatory failure. Supplemental oxygen therapy is fundamental, with delivery modalities ranging from nasal cannula to high-flow oxygen systems, aimed at maintaining adequate oxygenation both at rest and during exertion. It has been shown to improve exercise tolerance, reduce pulmonary hypertension risk, and support optimal growth and neurodevelopment [32].

For children with progressive ventilatory insufficiency or those unable to maintain oxygenation with supplemental oxygen alone, the use of non-invasive ventilation (NIV) techniques—such as continuous positive airway pressure (CPAP) or bi-level positive airway pressure (BiPAP)—can provide ventilatory support, reduce work of breathing, and improve sleep quality. NIV facilitates alveolar recruitment, reduces fatigue, and may prevent progression to invasive mechanical ventilation [33].

In advanced cases or as a bridge to transplantation, invasive mechanical ventilation may be employed. The decision to escalate support involves careful assessment of ventilatory drive, oxygenation status, and the child’s overall condition, with goal-directed adjustments guided by serial clinical evaluations, blood gases, and lung function testing [28].

Monitoring disease progression and therapeutic response requires serial assessments using the same modalities—pulse oximetry, ABG analysis, and PFTs—administered at regular intervals. Changes in oxygen requirements, lung volumes, or gas exchange parameters inform therapeutic adjustments, escalation of ventilation support, or reevaluation of treatment goals [32,33,34].

Overall, addressing chronic hypoxemia and ventilatory failure is a cornerstone of comprehensive pediatric ILD management. Early and proactive intervention improves functional status, decreases complication risks such as pulmonary hypertension, and supports growth and development, ultimately contributing to better long-term outcomes.

#### 3.1.5. Aspiration and Gastroesophageal Reflux Traits

Aspiration and gastroesophageal reflux (GER) are prevalent and significant traits in chILD, often acting as perpetuating factors that exacerbate inflammation, promote further lung injury, and complicate disease management. In many children with ILD, reflux can be silent or manifest with subtle symptoms, yet its role in propagating airway and parenchymal injury makes diagnosis and targeted treatment crucial [35,36,37].

The diagnostic evaluation of GER and aspiration involves a combination of clinical history, symptom assessment, and specialized testing. A detailed history should probe for frequent vomiting, regurgitation, feeding difficulties, respiratory symptoms exacerbated after meals, or unexplained chronic cough and recurrent pneumonia. Objective investigations include pH impedance testing, which measures esophageal acid exposure and detects non-acid reflux episodes, and videofluoroscopic swallow studies (VFSS)—also known as modified barium swallow tests—that assess swallowing function, airway protection, and potential aspiration during different feeding maneuvers [38,39].

In some cases, additional assessments such as esophageal manometry provide information about motility disorders, while radionuclide studies or salivagram can evaluate for silent aspiration. Laboratory assessments, including inflammatory markers and food allergy testing, help identify contributory factors such as allergies or hypersensitivity that may exacerbate reflux symptoms [40].

Management strategies focus on controlling reflux and minimizing aspiration, thereby reducing ongoing injury. Medical therapies include proton pump inhibitors (PPIs) or H2-receptor antagonists to decrease gastric acid production, although their role in preventing aspiration-related injury is debated and may be limited when non-acid reflux predominates. Prokinetic agents, such as metoclopramide or domperidone, can enhance esophageal motility and gastric emptying but are used cautiously due to potential side effects [41].

For children with documented or strongly suspected aspiration, secondary interventions may include speech and language therapy aimed at improving bolus control, swallowing safety, and respiratory coordination. Behavioral modifications, feeding techniques, and positioning strategies are emphasized to reduce aspiration risk [42].

In certain cases, especially when medical therapy fails or anatomical abnormalities are identified (e.g., severe GER), surgical interventions like fundoplication can be considered to mechanically prevent reflux. Additionally, addressing contributory factors such as allergies, eosinophilic esophagitis, or motility disorders is vital for comprehensive care [39,40,41].

Emerging approaches focus on refined diagnostic methods, targeted pharmacologic agents, and multidisciplinary care involving gastroenterology, pulmonology, and speech therapy teams. Recognizing and managing aspiration and GER as treatable traits can significantly attenuate ongoing inflammation, improve pulmonary outcomes, and enhance overall quality of life for children with ILD.

#### 3.1.6. Growth and Nutritional Deficits

Growth impairment and nutritional deficits are among the most common and impactful treatable traits in children with progressive or severe interstitial lung disease. Chronic pulmonary pathology, increased energy expenditure from labored breathing, malabsorption, feeding difficulties, and systemic inflammation contribute to failure to thrive, weight loss, and micronutrient deficiencies. These deficits not only impair physical development but also affect immune function, neurocognition, and overall quality of life, potentially accelerating disease progression [43].

Assessment begins with a comprehensive nutritional evaluation, including detailed anthropometric measurements—such as weight, height, head circumference (in infants), and body mass index (BMI)—compared to age- and sex-matched normative data. Growth velocity and pattern over time provide critical information about nutritional status. Dietary reviews, including detailed food intake assessments and feeding history, help identify deficiencies, inadequate caloric intake, or problematic feeding behaviors. Laboratory evaluations may include assessments of vitamins, minerals, hemoglobin levels, and markers of malabsorption or systemic inflammation [44].

Children with ILD often require individualized nutritional strategies tailored to their specific needs. These may include calorie-dense formulas, fortified feeds, and the use of supplemental nutrition support such as enteral feeding through nasogastric or gastrostomy tubes when oral intake is inadequate or unsafe. In some cases, micronutrient supplementation is necessary, especially for deficiencies in iron, vitamin D, zinc, or other key nutrients essential for growth, immune function, and tissue repair [45].

Optimizing nutrition can have profound effects on disease trajectory. Adequate caloric and nutrient intake supports immune resilience, promotes lung growth, and enhances response to therapy. Early intervention prevents further deterioration of growth parameters and reduces the risk of complications such as osteoporosis, muscle weakness, and developmental delays [46].

Given the potential for systemic inflammation and metabolic dysfunction to exacerbate chILD, the role of therapies targeting insulin resistance warrants consideration. While direct evidence examining the impact of insulin-sensitizing medications (such as metformin, GLP-1 agonists, or SGLT2 inhibitors) on the progression of chILD is currently lacking, insights can be gleaned from related conditions. For example, studies in adults with chronic lung diseases and co-existing diabetes have suggested that improved glycemic control may correlate with better respiratory outcomes. Furthermore, in cystic fibrosis, where CF-related diabetes is a common comorbidity, interventions targeting insulin resistance might indirectly benefit lung health by mitigating systemic inflammation and optimizing nutritional status. However, further research is needed to determine whether such interventions have a direct or indirect impact on lung function, inflammation, or disease progression in children with chILD, particularly those with evidence of insulin resistance or metabolic syndrome [47,48,49]

Multidisciplinary care involving dietitians, pulmonologists, and speech therapists (for swallowing assessment) is vital. Regular monitoring with serial anthropometry and dietary assessments enables clinicians to adjust nutritional plans proactively. Education and family support are equally important to ensure adherence and address challenges related to feeding and nutrition in this vulnerable population [50].

**Figure 1:**
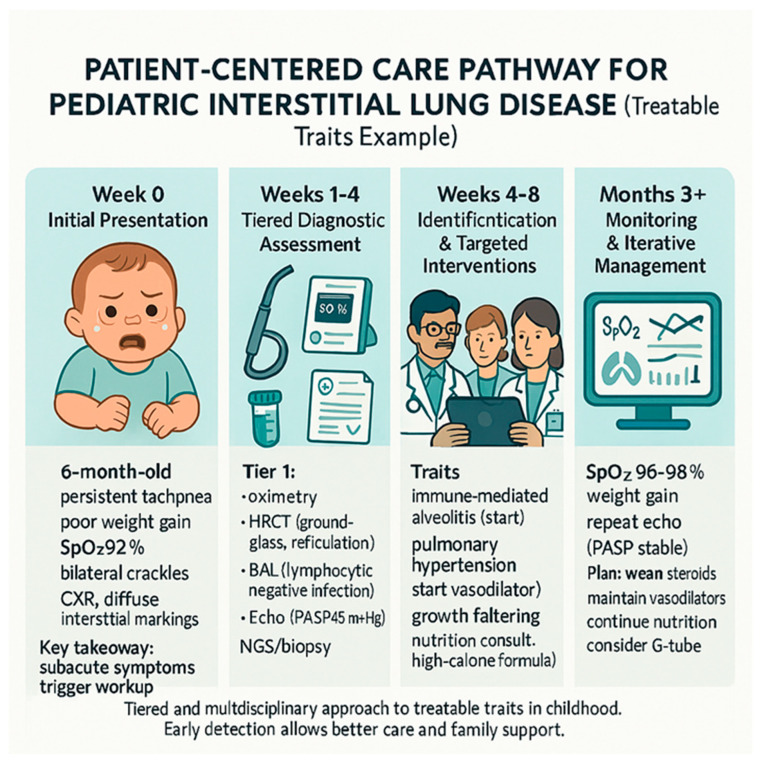
Illustrative patient-centered care pathway demonstrating tiered assessment, trait identification, targeted intervention, and iterative monitoring in a child with diffuse lung disease. This schematic visualizes the practical application of the treatable traits framework in a typical clinical scenario.

Figure 1 provides an example of how the treatable traits framework is applied in a real-world clinical scenario, highlighting the iterative process of assessment, diagnosis, and management in a child with diffuse lung disease. In conclusion, recognizing and addressing growth and nutritional deficits as treatable traits can significantly improve overall health outcomes, facilitate better response to pulmonary therapies, and improve the child’s potential for normal development and quality of life (Table 1).

### 3.2. Emerging and Environmental Traits

Beyond the classic biological and physiologic traits, there is increasing recognition of other modifiable factors that can influence the course of pediatric interstitial lung disease. These emerging and environmental traits often interact with primary disease drivers, potentially exacerbating lung injury or impeding recovery. Addressing these traits requires a multidisciplinary, holistic approach that considers external exposures, lifestyle factors, and broader health influences [51].

One such trait involves environmental exposures—including air pollution, passive cigarette smoke, occupational pollutants, and allergen inhalation—which can worsen inflammation and fibrosis. Children living in urban or industrial areas may encounter higher pollutant burdens, leading to increased airway reactivity, immune activation, and progressive lung damage. Identifying relevant exposures through detailed environmental histories and, where feasible, biomonitoring aids in establishing causality. Management primarily involves education about exposure avoidance, improving indoor air quality, and advocating for environmental health policies to reduce pollution burden [52].

Another important emerging trait is sleep-disordered breathing, which includes obstructive sleep apnea (OSA) and hypoventilation. These conditions may develop due to altered airway anatomy, neuromuscular weakness, or restriction from lung disease itself. Sleep studies (polysomnography) are crucial for diagnosis, as untreated sleep apnea can lead to intermittent hypoxemia, increased pulmonary pressures, and impaired growth. Management includes positive airway pressure therapy, positional strategies, and addressing contributing factors such as adenotonsillar hypertrophy or obesity [53,54].

Psychosocial and behavioral factors are also increasingly recognized as modifiable traits, impacting adherence to complex therapies such as inhaled medications, nutrition, or physiotherapy. Psychological support and family-centered counseling can improve engagement and overall health outcomes [55].

Lastly, recent research emphasizes the influence of systemic inflammation and metabolic health, such as obesity, insulin resistance, or dyslipidemia, which may exacerbate disease progression or impair recovery. Lifestyle modifications, including physical activity, nutritional optimization, and weight management, form part of a holistic management strategy.

Addressing these environmental and emerging traits offers an opportunity to optimize overall health, reduce additional inflammatory stimuli, and improve disease trajectory. Further research is needed to elucidate these traits’ mechanistic contributions and develop tailored interventions within a pediatric ILD management framework.

### 3.3. Treatable Traits in Pediatric Diffuse Lung Disease: A Practical, Tiered Approach

**Figure 2:**
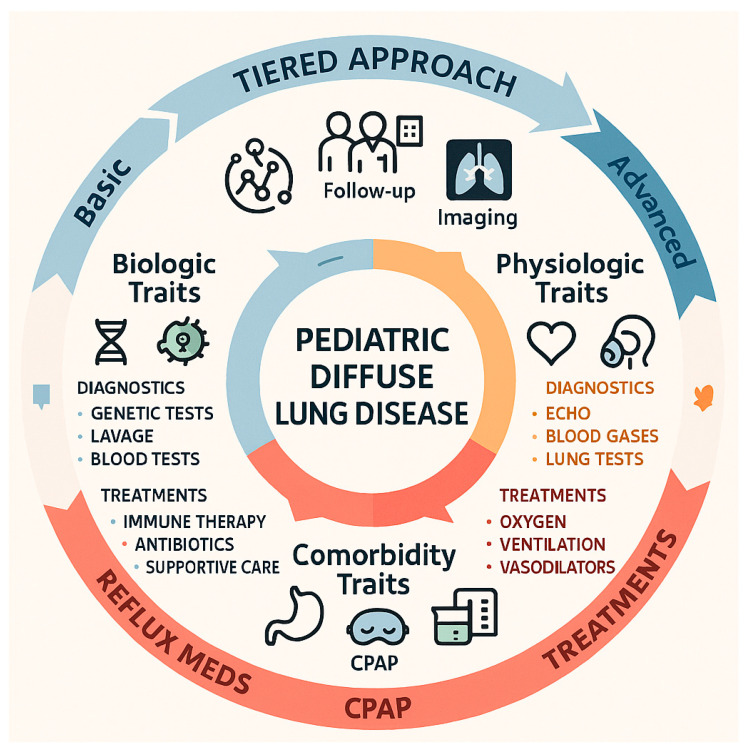
This figure presents a streamlined framework for the identification and management of treatable traits in children diagnosed with pediatric diffuse lung disease (DLD). This figure presents a streamlined model for identifying and managing treatable traits in pediatric diffuse lung disease. It organizes traits into three domains—biologic, physiologic, and comorbid—each linked to relevant diagnostics and therapies. A tiered diagnostic pathway guides clinical evaluation from basic to advanced testing, while multidisciplinary collaboration and outcome monitoring ensure comprehensive, individualized care.

Pediatric diffuse lung disease presents a recurring set of clinically actionable features—treatable traits—that transcend traditional diagnostic labels and can be systematically sought to guide precision care. Biologic traits include genetic surfactant pathway defects (SFTPB, SFTPC, ABCA3 and others) that often present in infancy and mandate early comprehensive genetic testing because identification informs prognosis, family counseling, transplant planning and eligibility for emerging molecular therapies; immune dysregulation manifesting as lymphocytic alveolitis, autoantibody-associated pneumonitis, or monogenic immune disorders is suggested by subacute deterioration, bronchoalveolar lavage lymphocytosis or positive serologies and frequently responds to corticosteroids and steroid-sparing immunomodulators when treated promptly; infectious and colonization traits—persistent viral, atypical bacterial or fungal infection—require targeted microbiologic workup and directed antimicrobial or airway clearance strategies to reduce inflammatory burden [56]. Physiologic traits such as pulmonary hypertension, chronic hypoxemia, and ventilatory insufficiency are readily identified by systematic screening (echocardiography, pulse oximetry, arterial blood gases and serial pulmonary function testing) and are amenable to specific interventions including pulmonary vasodilators, supplemental oxygen and non-invasive ventilation, which can change clinical trajectory if instituted early [57]. Comorbidity traits including aspiration and gastroesophageal reflux, sleep-disordered breathing, and growth or nutritional deficits are common contributors to ongoing lung injury; they are often detectable by pH-impedance monitoring, videofluoroscopic swallow studies, polysomnography and anthropometric assessment and are modified by medical, surgical and rehabilitative strategies such as anti-reflux measures, feeding therapy, positive airway pressure and targeted nutritional support [58]. Practically, trait identification should follow a tiered diagnostic approach—baseline clinical assessment and imaging, intermediate testing (pulmonary function, bronchoscopy with lavage, echo, reflux studies), and advanced investigations (next-generation genetic panels, lung biopsy, right-heart catheterization)—with multidisciplinary prioritization of traits by reversibility and urgency; recognizing that many traits coexist, clinicians must balance simultaneous interventions while acknowledging that pediatric-specific evidence for several targeted therapies remains limited and requires prospective study (Figure 2).

### 3.4. Implementation and Future Directions

Successfully integrating the treatable traits approach into routine pediatric respiratory care necessitates setting clear, pragmatic goals coupled with strategic steps designed to operationalize the concept and ensure sustainable impact. The overarching goal is to promote early and precise identification of key treatable traits using standardized, tiered diagnostic pathways, thereby facilitating timely, targeted interventions that can alter disease trajectories and improve long-term outcomes. To accomplish this, future strategies should focus on developing and validating clinically applicable diagnostic algorithms that incorporate specific triggers—such as persistent hypoxemia, unexplained radiological patterns, abnormal bronchoalveolar lavage cellular profiles, or evidence of pulmonary hypertension—that guide clinicians through stepwise testing. These algorithms must balance sensitivity and specificity and be adaptable to various healthcare settings, including resource-limited environments [6,15]. Establishing multidisciplinary trait teams is essential, bringing together pediatric pulmonologists, geneticists, immunologists, cardiologists, nutritionists, speech therapists, and other specialists. These teams should operate under shared protocols that prioritize traits by reversibility, urgency, and potential impact, with clearly defined time targets for intervention. A key future goal is the creation of integrated data platforms—national or international registries—that classify children by dominant traits, systematically record diagnostic tests, treatments, adverse events, and outcomes. The data collected will inform evidence-based guidelines and support pragmatic clinical trials designed to evaluate trait-targeted therapies efficiently, with enrollment based on phenotype rather than rare diagnoses [17]. Additionally, leveraging advanced health informatics—including decision support tools integrated into electronic health records—and telemedicine platforms will be crucial to broaden access, support clinician decision-making, and facilitate family engagement. Priorities for future research include validating novel, noninvasive biomarkers for early trait detection, conducting pediatric-specific trials for therapies commonly used in adult ILD but untested in children, and exploring gene–environment interactions for early prevention strategies. Addressing barriers such as limited pediatric data, resource variability, and health inequities will require collaborative efforts among clinicians, researchers, industry, and policymakers [59]. Ultimately, the goal is to establish an evidence-guided, scalable, and sustainable framework that makes trait-based pediatric respiratory care a standard, reproducible feature of future practice, with continuous refinement driven by ongoing research and real-world data.

## 4. Discussion

The findings outlined in this review provide compelling evidence that a treatable traits approach holds significant promise for advancing the management of pediatric interstitial lung disease (Table 2). By shifting focus from broad disease labels to specific, modifiable features, clinicians can adopt a more personalized, targeted strategy that has the potential to improve clinical outcomes, reduce unnecessary treatments, and facilitate early intervention.

One of the most salient points emerging from this synthesis is that many traits traditionally considered fixed or diagnostic labels—such as genetic mutations or fibrotic changes—may, in fact, be circumvented or mitigated through early and precise therapeutic targeting. For instance, genetic and surfactant traits, although historically viewed as inexorable or solely supportive in management, are now increasingly amenable to early diagnosis through next-generation sequencing and functional studies. Recognizing such traits early may open pathways to novel therapies, influence family counseling, and inform prognosis more accurately [7,8,10,15].

Similarly, traits related to immune dysregulation, when identified promptly via BAL cellular profiles or serological markers, enable targeted immunomodulation that could arrest or slow disease progression. This concept aligns with broader moves in pediatric rheumatology and immunology, where early, trait-based intervention has yielded favorable outcomes. Nevertheless, robust evidence supporting specific interventions in pediatric ILD remains limited, highlighting a crucial gap that future research should address [16,17,60].

Pulmonary vascular and hypoxemia-related traits, often overlooked until advanced disease occurs, have profound impacts on prognosis. Early detection via screening echocardiography and serial monitoring can facilitate timely use of vasodilators and oxygen therapy, respectively. Evidence, although primarily extrapolated from adult data, suggests that early treatment may improve functional capacity and survival. Yet, clinical trials dedicated to pediatric populations are necessary to refine these approaches, determine optimal timing, and evaluate long-term safety [28,30,31].

Furthermore, environmental and lifestyle factors—such as exposure to pollutants, sleep-disordered breathing, and nutritional status—represent modifiable traits that are often underappreciated but carry significant implications for disease evolution. Addressing these traits requires multidisciplinary collaboration and integration of social, behavioral, and environmental health strategies into standard care [61].

Despite the enthusiasm for the treatable traits model, substantial challenges remain. The paucity of pediatric-specific data on many interventions hampers widespread adoption. Most evidence derives from adult ILD or smaller pediatric case series, emphasizing the need for dedicated pediatric research initiatives, including multicenter registries, prospective cohort studies, and pragmatic trials. Additionally, variability in healthcare access, diagnostic resources, and expertise complicates implementation, particularly in resource-limited settings [62].

The broad implications extend beyond individual patient management. Implementing trait-based models can streamline research efforts by enabling more homogeneous patient grouping for clinical studies, fostering international collaborations, and facilitating the development of consensus guidelines tailored specifically for pediatric ILD.

Looking ahead, several priorities emerge. First, validating reliable, non-invasive biomarkers that can rapidly identify and monitor treatable traits is essential for timely intervention. Second, investing in the development of targeted therapies—including gene-based and molecular approaches—may revolutionize treatment options for genetic and immune-mediated traits. Third, creating standardized protocols for trait assessment and management algorithms will support consistent implementation and improve inter-center comparability [63].

Finally, education and training remain critical, ensuring that pediatric pulmonologists, allergists, immunologists, and allied health professionals are equipped to recognize and treat the breadth of traits. Incorporating a family-centered approach, addressing psychosocial factors, and engaging caregivers in trait management plans will also strengthen outcomes.

It is important to acknowledge several limitations of this review. First, this work presents a narrative synthesis of the literature and expert opinion, rather than a systematic review or meta-analysis; therefore, the comprehensiveness and generalizability of findings may be limited. Second, the evidence base for applying the ‘treatable traits’ framework to pediatric ILD is still developing. Many of the management strategies discussed are based on extrapolation from adult ILD studies, and further pediatric-specific research is needed to validate their efficacy and safety in children. Third, chILD encompasses a heterogeneous group of rare disorders, which presents challenges for conducting large-scale clinical trials and generating robust evidence. Finally, it’s crucial to recognize that the treatable traits framework, as applied to pediatric ILD, remains largely conceptual. Further research is necessary to establish the clinical utility, refine the identification of key traits, and develop evidence-based algorithms for trait-based management.

## 5. Conclusions

The treatable traits approach offers a promising pathway toward personalized and more effective management of pediatric interstitial lung disease. By systematically identifying and targeting discrete, modifiable features—ranging from genetic mutations to environmental exposures—clinicians can optimize outcomes, prevent disease progression, and improve quality of life in affected children. While the treatable traits approach offers a promising pathway toward personalized care, it is crucial to acknowledge practical limitations to its real-world application in pediatric ILD. These include the scarcity of validated biomarkers, variability in access to diagnostic resources, uncertainty regarding treatment efficacy due to limited pediatric-specific clinical trials, challenges related to multidisciplinary collaboration, and potential cost considerations. Overcoming these barriers will be essential to realize the full potential of this approach and ensure equitable access to improved care for all children with ILD.

## Figures and Tables

**Table 1:** Summary of Core Treatable Traits in Pediatric Interstitial Lung Disease.

Trait Category	Diagnostic Tools	Key Interventions/Management Options	Evidence Level/Notes
**Genetic & Surfactant Disorders**	Genetic testing (next-generation sequencing), EM, IHC	Supportive care, lung transplantation, emerging gene therapies	Limited pediatric data; ongoing trials
**Immune-Mediated Inflammation**	BAL cellular analysis, immune serologies, biopsy	Corticosteroids, immunomodulators (e.g., MMF, azathioprine)	Variable response; early detection improves outcomes
**Pulmonary Hypertension**	Echocardiography, cardiac catheterization	Vasodilators (PDE5 inhibitors, endothelin antagonists), oxygen therapy	Extrapolated from adult data; early detection critical
**Hypoxemia & Ventilatory Failure**	ABG, PFTs, pulse oximetry, clinical assessment	Supplemental oxygen, NIV, mechanical ventilation	Serial monitoring essential
**Aspiration & GER**	pH impedance, VFSS, clinical history	Medical therapy (PPIs), speech therapy, surgery (fundoplication)	Individualized; multimodal approach preferable
**Growth & Nutritional Deficits**	Anthropometry, dietary review, labs	Enteral feeding, nutritional supplements, multidisciplinary support	Impact on overall prognosis; early intervention beneficial

**Table 2:** Diagnostic and Therapeutic Algorithm for Pediatric Interstitial Lung Disease Traits.

Step	Assessment/Question	Diagnostic Approach	Management Step
1	Does the child have low oxygen levels at rest or during activity?	Use pulse oximetry and arterial blood gas analysis	Provide supplemental oxygen as needed
2	Are there indications of immune system dysregulation?	Perform bronchoalveolar lavage analysis, immune serology testing, and, if necessary, lung biopsy	Initiate appropriate immune-suppressing or modulating therapies
3	Is pulmonary hypertension suspected or confirmed?	Conduct echocardiography; confirm with cardiac catheterization if indicated	Start medications to dilate the pulmonary blood vessels and carefully monitor heart function
4	Is there evidence of aspiration or reflux?	Perform pH impedance testing and swallowing studies with contrast	Use medications to reduce stomach acid, and involve speech therapy to improve swallowing and feeding techniques
5	Are there concerns about poor growth or nutritional deficits?	Conduct anthropometric measurements and dietary assessment, and perform laboratory tests as needed	Provide nutritional support such as special diets or tube feeding to promote growth
6	Has a genetic disorder affecting lung surfactant or surfactant protein genes been identified?	Use genetic testing, lung tissue examination with electron microscopy, and protein staining tests	Supportive treatments, and consider lung transplantation in severe cases

## Data Availability

Not applicable.

## Abbreviations

The following abbreviations are used in this manuscript:

ABG	Arterial blood gas(s)
ALT	Alanine transaminase
ABCA3	ATP-binding cassette transporter A3 (gene symbol)
BAL	Bronchoalveolar lavage
BiPAP	Bi-level positive airway pressure
BMI	Body mass index
CRP	C-reactive protein
CT	Computed tomography
DLCO	Diffusing capacity for carbon monoxide
EM	Electron microscopy
FVC	Forced vital capacity
GER	Gastroesophageal reflux
HLA	Human leukocyte antigen
HRCT	High-resolution computed tomography
IFN	Interferon
IL	Interleukin
IHC	Immunohistochemistry
ICU	Intensive care unit
MMF	Mycophenolate mofetil
NGS	Next-generation sequencing
NK	Natural killer (cells)
NIV	Non-invasive ventilation
OSA	Obstructive sleep apnea
PFT	Pulmonary function test(s)
PDE5	Phosphodiesterase type 5
